# Antiproliferative Activity of *Buddleja saligna* (Willd.) against Melanoma and *In Vivo* Modulation of Angiogenesis

**DOI:** 10.3390/ph15121497

**Published:** 2022-11-30

**Authors:** Danielle Twilley, Velaphi C. Thipe, Navneet Kishore, Pierce Bloebaum, Catarina Roma-Rodrigues, Pedro V. Baptista, Alexandra R. Fernandes, Mamoalosi A. Selepe, Lenka Langhansova, Kattesh Katti, Namrita Lall

**Affiliations:** 1 Department of Plant and Soil Sciences, Faculty of Natural and Agricultural Sciences, University of Pretoria, Pretoria 0002, South Africa; 2 Department of Radiology, Institute of Green Nanotechnology, School of Medicine, University of Missouri, Columbia, MO 65212, USA; 3 Sinclair Research Centre, Auxvasse, MO 65231, USA; 4 UCIBIO-Applied Molecular Biosciences Unit, Departamento de Ciências da Vida, Faculdade de Ciências e Tecnologia, Universidade NOVA de Lisboa, 2829-516 Caparica, Portugal; 5 Department of Chemistry, Faculty of Natural and Agricultural Sciences, University of Pretoria, Pretoria 0002, South Africa; 6 Laboratory of Plant Biotechnologies, Czech Academy of Sciences, Institute of Experimental Botany, 165 00 Prague, 6-Lysolaje, Czech Republic; 7 University of Missouri Research Reactor (MURR), University of Missouri, Columbia, MO 65212, USA; 8 Department of Physics and Astronomy, University of Missouri, Columbia, MO 65211, USA; 9 School of Natural Resources, University of Missouri, Columbia, MO 65211, USA; 10 College of Pharmacy, JSS Academy of Higher Education and Research, Mysuru 570015, India; 11 Bio-Tech Research and Development Institute, The University of the West Indies, Mona, Kingston 7, Jamaica, West Indies

**Keywords:** *Buddleja saligna*, melanoma, antiproliferative activity, angiogenesis, ex ovo YSM

## Abstract

Melanoma cells secrete pro-angiogenic factors, which stimulates growth, proliferation and metastasis, and therefore are key therapeutic targets. *Buddleja saligna* (BS), and an isolated triterpenoid mixture (DT-BS-01) showed a fifty percent inhibitory concentration (IC_50_) of 33.80 ± 1.02 and 5.45 ± 0.19 µg/mL, respectively, against melanoma cells (UCT-MEL-1) with selectivity index (SI) values of 1.64 and 5.06 compared to keratinocytes (HaCat). Cyclooxygenase-2 (COX-2) inhibition was observed with IC_50_ values of 35.06 ± 2.96 (BS) and 26.40 ± 4.19 µg/mL (DT-BS-01). BS (30 µg/mL) significantly inhibited interleukin (IL)-6 (83.26 ± 17.60%) and IL-8 (100 ± 0.2%) production, whereas DT-BS-01 (5 µg/mL) showed 51.07 ± 2.83 (IL-6) and 0 ± 6.7% (IL-8) inhibition. Significant vascular endothelial growth factor (VEGF) inhibition, by 15.84 ± 4.54 and 12.21 ± 3.48%, respectively, was observed. In the ex ovo chick embryo yolk sac membrane assay (YSM), BS (15 µg/egg) significantly reduced new blood vessel formation, with 53.34 ± 11.64% newly formed vessels. Silver and palladium BS nanoparticles displayed noteworthy SI values. This is the first report on the significant anti-angiogenic activity of BS and DT-BS-01 and should be considered for preclinical trials as there are currently no US Food and Drug Administration (FDA) approved drugs to inhibit angiogenesis in melanoma.

## 1. Introduction

Over the past decade, the number of skin cancer cases has increased globally. In the US alone, the number of annually diagnosed invasive melanoma cases increased by 44% between 2011–2021, whereas the diagnosis and treatment of non-melanoma skin cancer increased by 77% between 1994–2014 [1]. According to incidence data from the World Bank for Cancer, non-melanoma skin cancer resulted in 1,042,056 (6.08 per 100,000) cases in 2018, whereas cutaneous melanoma resulted in 287,723 (1.68 in 100,000) cases, ranking 5th and 21st in the world’s most prevalent cancers, respectively [2]. 

Angiogenesis, the process by which new blood vessels are formed from existing ones, is one of the major hallmarks of cancer [3]. Tumor cells rely on angiogenesis for the continued supply of oxygen and nutrients to progress, survive and metastasize. For tumor cells to metastasize beyond their primary site, it is imperative that these cells produce pro-angiogenic factors which stimulate local angiogenesis, thereby providing sufficient nutrients for growth and proliferation, and a route to spread to different parts of the body. Inhibiting angiogenesis is therefore a potential therapeutic target for cancer therapy. In adulthood, angiogenesis is activated for physiological functions such as wound healing. Angiogenesis also plays a pivotal role in cancer progression, requiring continuous activation for the survival of the tumor cells [3,4]. Although it is hypothesized that angiogenesis inhibitors may not result in significant side effects due to the limited role in normal physiological processes in adulthood, the actual impact still needs to be fully evaluated. 

Studies as early as 1966, showed that cutaneous melanoma triggers pro-angiogenic activity. This was confirmed by Hubler and Wolf (1976) [5], who observed that after 48 h of melanoma transplantation into the cheek pouches of hamsters, beading of pre-existing vessels occurred, followed by the presence of budding of capillaries after 3 days to form vessel sprouts and finally after 4–6 days, the tumor was penetrated by new vessels to form a vascular network. Warren and Shubik (1996) [6] observed a particular vascular pattern that developed in growing melanoma cells transplanted into the cheek pouch of Syrian hamsters. Melanoma, which develops in melanocytes and often manifests as a nevus/mole, requires the formation of new blood vessels to progress. Nevi can transform into abnormal nevi, known as dysplastic nevi, which follow a radial growth phase pattern with cells spreading horizontally across the skin’s epidermis layer. Thereafter, cells are able to follow a vertical growth phase pattern and penetrate the dermis. During this transformation from radial to vertical growth phase, growth factors are produced that stimulate angiogenesis, and therefore facilitates metastasis. Metastasis is also able to occur through the lymphatic system. Once tumor cells have migrated to lymphatic vessels, they infiltrate the lymph nodes, providing tumor cells with a pathway to the lungs, brain and liver [7].

Angiogenesis in melanoma cells is activated by the secretion of growth factors by the tumor cells, which therefore may be looked upon as promising targets for the potential inhibition of angiogenesis. During the transformation of a dysplastic nevus from the radial growth phase to the vertical growth phase, melanocytes produce a large quantity of vascular endothelial growth factor (VEGF) stimulating the growth of new blood vessels, which is continued throughout the growth of the new blood vessels [7]. According to Bar-Eli (1999) [8], interleukin-8 (IL-8) serum levels were increased in patients with melanoma compared to patients without melanoma and the levels of IL-8 increased as the melanoma progressed [9,10]. Both melanoma and endothelial cells secrete IL-8, which has been demonstrated to enhance melanoma growth and migration [7], as well as angiogenesis, metastasis and vascular permeability [11]. Interleukin-6 (IL-6), which plays a major role in cancer progression, inhibits apoptosis in tumor cells and promotes angiogenesis [12]. Metastatic melanoma cells have been reported to exhibit elevated expression levels of IL-6 [13,14]. Cyclooxygenase-2 (COX-2), an inducible enzyme which is upregulated in several melanoma cell lines, contributes to metastasis of melanoma [15]. It has been shown to upregulate VEGF expression through the protein kinase C pathway, thus promoting angiogenesis in a variety of tumor cells [16]. 

Despite numerous clinical studies involving potential angiogenesis inhibitors, there are presently no anti-angiogenic drugs approved by the US Food and Drug Administration (FDA) for the treatment of melanoma. The FDA has authorized Bevacizymab (Avastin) for the treatment of several different types of cancer [17], however its use against angiogenesis in melanoma is currently still under investigation [4]. To address this greatly unmet need, the anti-angiogenic properties of plants, secondary metabolites and nanoformulations, are being investigated. Over the last three decades, Katti et al., have pioneered the utility of electron-rich phytochemicals as electron reservoirs in the transformation of metal salts, particularly of gold and silver, into phytochemical encapsulated nanoparticles [18,19,20,21]. This approach to green nanotechnology has resulted in the development of tumor specific gold nanoparticles [19,21]. Recently, the clinical translation of phytochemical conjugated gold nanoparticles (AuNPs) for treating various neoplastic diseases including breast and other cancers has been demonstrated [22]. Several studies have relied on the chorioallantoic membrane assay (CAM) to demonstrate the ability of nanoparticles to inhibit migration of cells and the formation of new blood vessels [23,24,25]. For example, NBTXR3 (Hensify), a novel tumor-agnostic hafnium oxide nanoparticle radio-enhancer, that has been activated with radiation, is used for the treatment of locally advances squamous cell carcinoma and other solid tumors [26,27]. Recently, the FDA authorized Tozinameran and mRNA-1273, two lipid nanoparticle drugs, for preventing COVID-19, demonstrating the importance of nanoformulations in precision and future therapeutics [28].

Drug discovery from natural resources remains a primary research focus due to the key role natural products have played in the treatment of human diseases. Often the discovery of drugs from natural resources has resulted in the identification of novel drugs which have unique mechanisms of action [29]. It has been estimated that more than 60% of anticancer drugs have been derived from natural resources [30], of which plants are the primary source. However, a key challenge when developing drugs from natural resources, remains the sustainable supply and use thereof. A major concern remains the overharvesting of plants in the wild and the unsustainable use of plants, where bulbs, roots and bark is harvested instead of leaves [31]. An example thereof, is the well-known anticancer drug Taxol™ that was developed from the bark of *Taxus brevifolia*, which later was found to be unsustainable and has since led to the semi-synthetic production of Taxol from the needles of *Taxus baccata* [32]. Conversely, in the current study, an indigenous South African plant, *Buddleja saligna* Willd, has been evaluated for its antiproliferative and anti-angiogenic activity which is a fast growing evergreen shrub, is widespread throughout South Africa, can grow up to 800 mm per year and can easily be grown from cuttings or seed [33]. In addition, significant activity was observed from the leaves and stems, further contributing towards its sustainable use.

The purpose of this study was to evaluate the in vitro antiproliferative and anti-angiogenic activity of an ethanolic extract prepared from the leaves and stems of *Buddleja saligna* (BS) and a compound mixture isolated from BS (DT-BS-01), to determine their potential use as therapeutic agents for the inhibition of melanoma angiogenesis and proliferation. The antiproliferative activity and mechanism of cell death (apoptosis) of malignant melanoma (UCT-MEL-1) cells and non-tumorigenic human keratinocytes (HaCat) were determined. The anti-angiogenic potential of BS and DT-BS-01 was assessed by evaluating their inhibitory activity against several pro-angiogenic factors, including vascular endothelial growth factor (VEGF), interleukin-6 and 8 (IL-6 and IL-8) as well as cyclooxygenase-2 (COX-2). Additionally, the ex ovo yolk sac membrane (YSM) assay was used to evaluate the in vivo potential to inhibit the formation of new blood vessels (angiogenesis).

## 2. Results

### 2.1. Antiproliferative Activity and Bio-Assay Guided Fractionation

**Figure 1:**
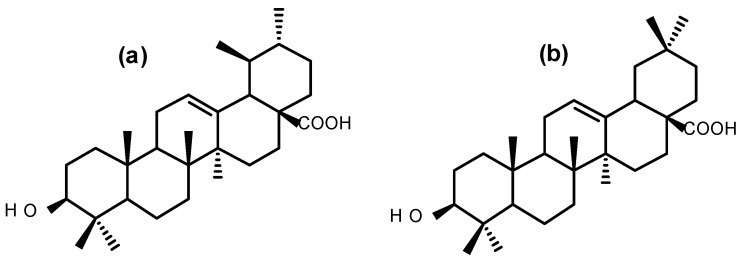
Chemical structures of (**a**) ursolic acid and (**b**) oleanolic acid.

The antiproliferative activity of BS against UCT-MEL-1 cells showed a fifty percent inhibitory concentration (IC_50_) of 33.80 ± 1.02 µg/mL. Against non-tumorigenic human keratinocytes (HaCat), an IC_50_ of 54.38 ± 8.55 was observed, and a selectivity index (SI) of 1.64, suggesting that BS was less toxic towards keratinocytes (SI > 1). The positive control, actinomycin D showed an IC_50_ value of 2.59 × 10^−^^3^ ± 4.85 × 10^−^^4^ and 5.57 × 10^−^^3^ ± 2.50 × 10^−^^4^ µg/mL against UCT-MEL-1 and HaCat cells, respectively, with an SI of 1.52 (Table 1). From the dichloromethane (DCM) partition, bio-assay guided fractionation yielded four major fractions (M1–M4), with M4 exhibiting the highest activity (Table 1). Fractionation of M4 resulted in the isolation of a triterpenoid mixture (DT-BS-01), with a promising SI value of 5.06, and IC_50_ values of 5.45 ± 0.19 (11.93 µM) and 27.59 ± 2.86 µg/mL (60.41 µM) against UCT-MEL-1 and HaCat cells, respectively (Table 1). The triterpenoid mixture DT-BS-01 was determined to be a mixture of two isomers, ursolic acid (UA) and oleanolic acid (OA) (Figure 1 and Figures S1–S5).

**Figure 2:**
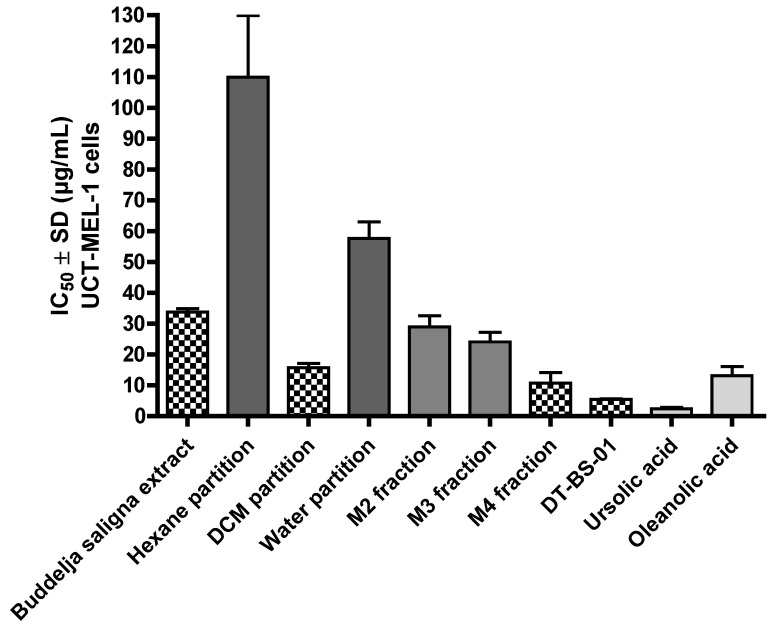
Fifty percent inhibitory concentration (IC_50_) values of *Buddleja saligna* ethanolic extract, partitions, major fractions and compounds isolated from *B. saligna* for a 72 h treatment period against human maligant melanoma cells (UCT-MEL-1) deteremined using XTT.

**Figure 3:**
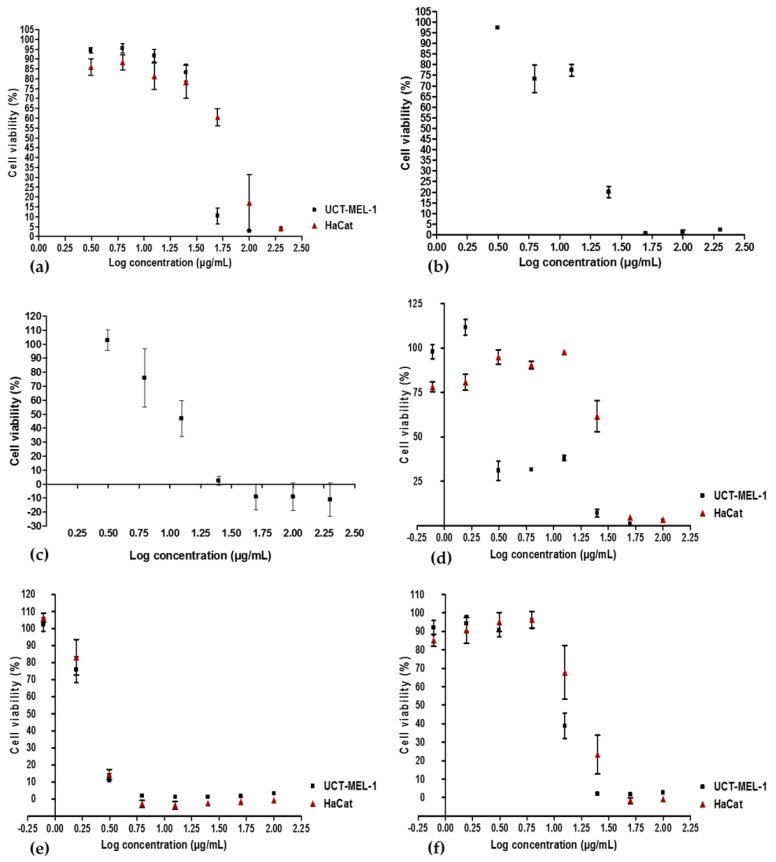
Dose—response curves depicting the antiproliferative activity of (**a**) *Buddleja saligna* ethanolic extract, (**b**) dichloromethane partition, (**c**) M4 major fraction, (**d**) DT-BS-01 triterpenoid mixture, (**e**) ursolic acid and (**f**) oleanolic acid against human malignant melanoma (UCT-MEL-1) cells and human keratinocytes (HaCat). Data shown are mean ± SEM (*n* = 3).

The antiproliferative activity of OA and UA was evaluated against UCT-MEL-1 and HaCat cells, with UA exhibiting an IC_50_ value of 2.31 ± 0.54 µg/mL (5.06 µM) and 2.32 ± 0.52 µg/mL (5.10 µM), respectively (SI: 1.01), and OA showed an IC_50_ value of 13.08 ± 3.03 µg/mL (28.64 µM) and 16.84 ± 1.32 µg/mL (36.87 µM), respectively (SI: 1.29). These findings showed that DT-BS-01 has a higher selectivity towards UCT-MEL-1 cells compared to non-tumorigenic keratinocytes. Moreover, DT-BS-01 showed a higher SI value than the positive control, actinomycin D (Figure 2 and Figure 3).

### 2.2. Apoptosis Detection-Microscopy

**Figure 4:**
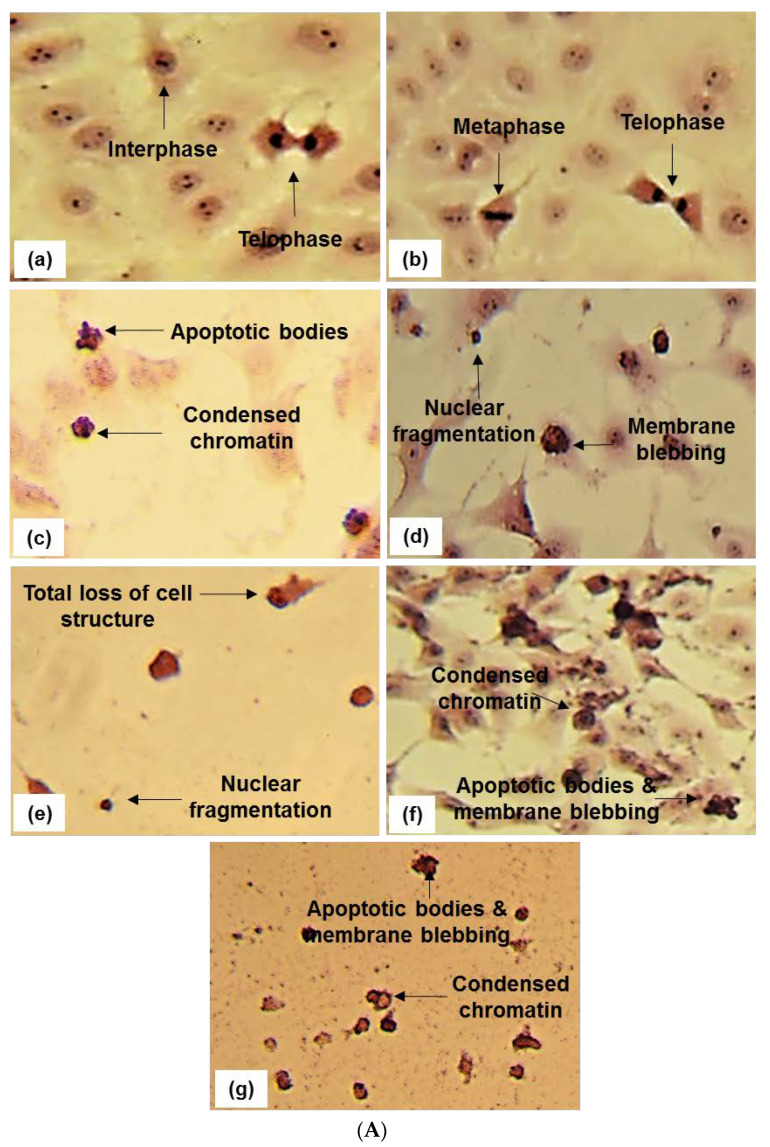

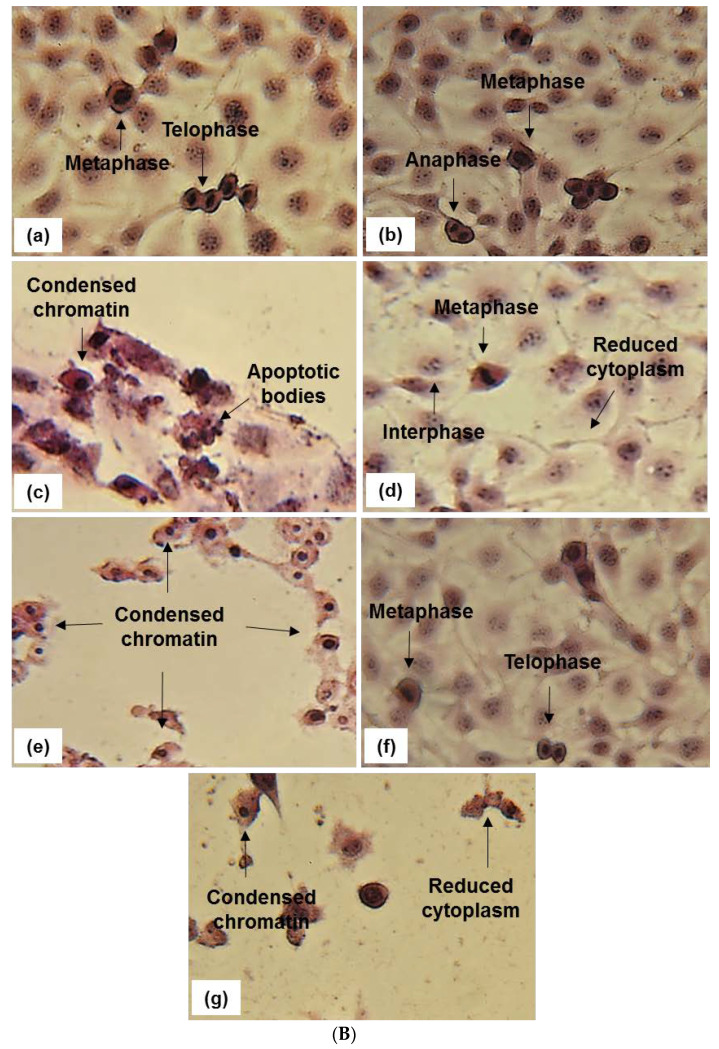
In (**A**) Hematoxylin and eosin staining (20 and 40× magnification) of UCT-MEL-1 cells after 48 h exposure to; (a) media (untreated) control, (b) 0.25% DMSO (vehicle) control, (c) 0.025 µg/mL actinomycin D (Act D) positive control, (d,e) *Buddleja saligna* extract (30 and 60 µg/mL) and (f,g) isolated triterpenoid mixture DT-BS-01 (5 and 20 µg/mL). Data are representative of one of two similar experiments. In (**B**) Hematoxylin and eosin staining (20 and 40× magnification) of HaCat cells after 48 h exposure to; (a) media (untreated) control, (b) 0.25% DMSO (vehicle) control, (c) 0.025 µg/mL actinomycin D (Act D) positive control, (d,e) *Buddleja saligna* extract (30 and 60 µg/mL) and (f,g) isolated triterpenoid mixture DT-BS-01 (5 and 20 µg/mL). Data are representative of one of two similar experiments.

The effect of BS and DT-BS-01 on the morphology of UCT-MEL-1 and HaCat cells was examined to determine whether cell death was mediated through apoptosis. Untreated cells and vehicle-treated control cells (0.25% DMSO) exhibited typical mitotic phases in both UCT-MEL-1 and HaCat cells (Figure 4). Actinomycin D displayed characteristic signs of apoptosis (apoptotic bodies, condensed chromatin, and membrane blebbing) in both cell lines. When treated with 30 µg/mL BS (corresponds to IC_50_ on UCT-MEL-1), UCT-MEL-1 cells exhibited an increase in apoptotic cells (fragmented nucleus, membrane blebbing, condensed chromatin) and a reduction in cells undergoing mitosis, while the majority of HaCat cells were observed in interphase. At 60 µg/mL BS (corresponds to 2 × IC_50_ on UCT-MEL-1 and approximate IC_50_ on HaCat), UCT-MEL-1 cells exhibited a complete loss of cell structure and low cell densities, while HaCat cells exhibited a loss of mitotic characteristics, reduced cell density and cells with condensed chromatin. HaCat cells treated with 5 µg/mL DT-BS-01 (IC_50_ on UCT-MEL-1) exhibited no signs of cell death, while UCT-MEL-1 cells exhibited apoptosis (condensed chromatin, apoptotic bodies and membrane blebbing). At 20 µg/mL DT-BS-01 (IC_50_ on HaCat), UCT-MEL-1 cells exhibited a complete loss of cell structures and apoptosis, comparable to what was observed in HaCat cells; however, the cytoplasm was not as reduced.

### 2.3. Cyclooxygenase-2 Inhibition

**Figure 5:**
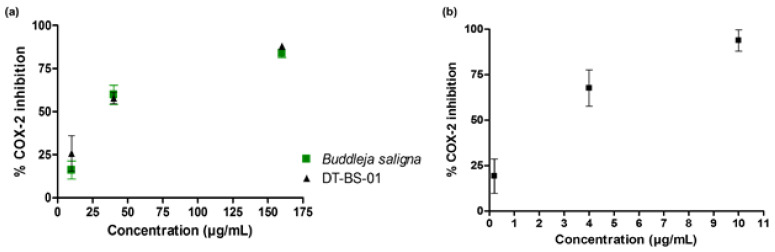
Dose—response curves of (**a**) *Buddleja saligna* (10, 40 and 160 µg/mL) and DT-BS-01 (10, 40 and 160 µg/mL) on COX-2 mediated PGE_2_ production compared to the positive control, (**b**) Ibuprofen (0.2, 4 and 10 µM). Data shown are mean ± SD (*n* = 3).

The potential of BS and DT-BS-01 to inhibit COX-2 was determined by measuring the concentration of prostaglandin E2 (PGE_2_). Both samples inhibited the production of PGE_2_ in a dose—dependent manner with IC_50_ values of 35.06 ± 2.96 and 26.40 ± 4.19 µg/mL for BS and DT-BS-01, respectively (Figure 5a). The activity was compared to the positive control, ibuprofen (IC_50_: 1.33 ± 0.70 µM/~0.27 µg/mL). At a concentration of 10 µg/mL, BS and DT-BS-01 showed 16.07 ± 5.27 and 25.73 ± 17.91% inhibition, respectively, while ibuprofen at 10 µM (~2.06 µg/mL) showed 93.82 ± 5.90% inhibition of COX-2. At an increased concentration of 160 µg/mL, the percentage inhibition of BS and DT-BS-01 increased to 83.84 ± 2.50 and 87.94 ± 1.85%, respectively (Figure 5a,b).

### 2.4. Cytokine Inhibition

**Figure 6:**
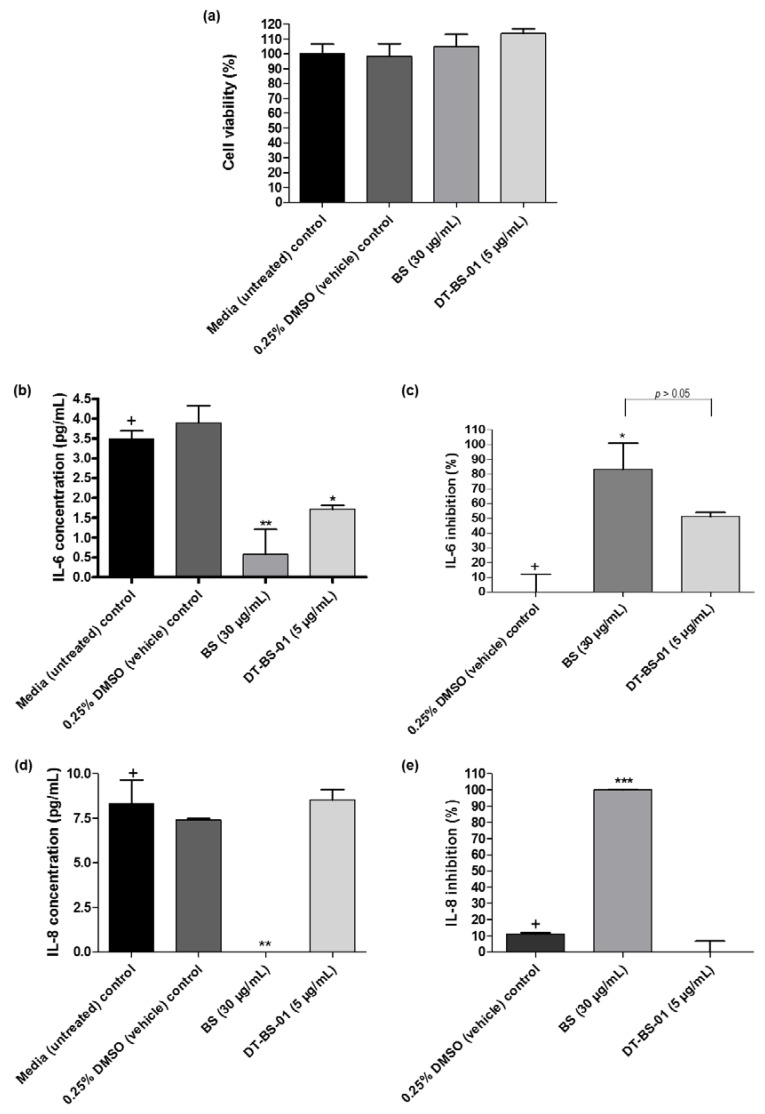
Quantification of human inflammatory cytokine production in UCT-MEL-1 cells after treatment with *Buddleja saligna* extract (BS: 30 µg/mL) and isolated triterpenoid mixture (DT-BS-01: 5 µg/mL) for 20 h. Controls included the 0.25% DMSO (vehicle) control and cells grown in medium (untreated). (**a**) Percentage cell viability, (**b**) concentration of IL-6 (pg/mL), (**c**) percentage IL-6 inhibition, (**d**) concentration of IL-8 (pg/mL) and (**e**) percentage IL-8 inhibition. Data was expressed as mean ± SD (*n* = 2). * *p* < 0.05, ** *p* < 0.01 and *** *p* < 0.001 indicates statistical significance when compared to the control (+). Statistical analysis was done using one-way ANOVA followed by Tukey’s Multiple Comparison Test.

Cell viability was evaluated to ascertain that inhibition of cytokine production was not due to cell death. When the viability of cells treated with the samples was compared to that of untreated cells, no statistical difference (*p* > 0.05) was observed (Figure 6a). No production of IL-1β, IL-10, IL-12p70 and TNF-α was detected in the PHA stimulated UCT-MEL-1 cells. However, IL-6 and IL-8 were produced. The difference in IL-8 and IL-6 production between the DMSO vehicle control and untreated control (growth media alone), was not significant (*p* > 0.05), suggesting that DMSO had no effect on IL-8 or IL-6 production in UCT-MEL-1 cells (Figure 6b,d). Both BS (*p <* 0.01) and DT-BS-01 (*p* < 0.05) significantly decreased IL-6 production (Figure 6b) by 83.26 ± 17.60 (*p <* 0.05) and 51.07 ± 2.83% (*p* > 0.05), respectively. However, inhibition by BS and DT-BS-01 compared to each other showed no significant difference (*p* > 0.05), suggesting that DT-BS-01 had a similar effect on IL-6 production as BS (Figure 6c). BS significantly decreased IL-8 production (*p* < 0.01) when compared to untreated cells, while DT-BS-01 had no effect (*p* > 0.05) (Figure 6d). When compared to the DMSO vehicle control, a percentage inhibition of 100 ± 0.2 (*p* < 0.001) and 0 ± 6.7% (*p* > 0.05) was observed for BS and DT-BS-01, respectively (Figure 6e).

### 2.5. VEGF Inhibition

**Figure 7:**
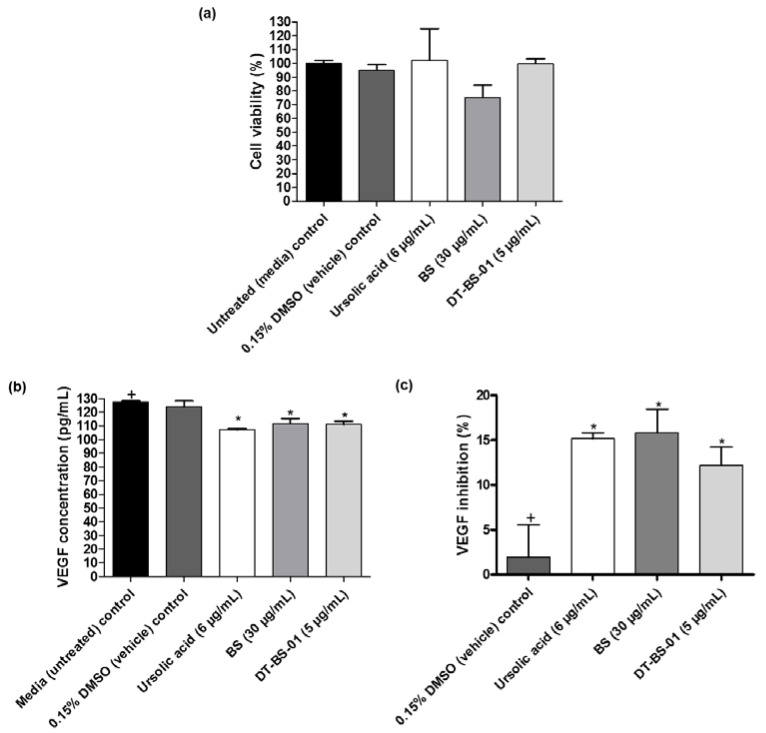
Quantification of VEGF in HaCat cells after treatment with BS (30 µg/mL) and DT-BS-01 (5 µg/mL) for 6 h. Controls included the positive control, ursolic acid (6 µg/mL), DMSO (0.15%) vehicle control and cells grown in medium (untreated). (**a**) Percentage cell viability, (**b**) vascular endothelial growth factor (VEGF) concentration (pg/mL) and (**c**) percentage VEGF inhibition. Data was expressed as mean ± SEM (*n* = 3). * *p* < 0.05 indicates statistical significance when compared to the control (+). Statistical analysis was done using one-way ANOVA followed by Dunnett’s Multiple Comparison Test.

In untreated UCT-MEL-1 cells, no VEGF was detected, suggesting that the cells did not actively produce VEGF. As such, HaCat cells were utilized to measure the VEGF levels in cell culture. Cell viability was evaluated after treatment with the samples to establish that VEGF inhibition was not caused by cell death. When the samples were compared to the viability of untreated cells, no statistical difference (*p* > 0.05) was observed (Figure 7a). Untreated cells expressed VEGF at a concentration of 127.50 ± 1.25 pg/mL, which was statistically similar (*p* > 0.05) to the 0.15% DMSO vehicle control (123.75 ± 7.81 pg/mL), suggesting that DMSO had no effect on VEGF production (Figure 7b). Additionally, VEGF measured after ursolic acid (positive control), BS and DT-BS-01 treatments were significantly different (*p* < 0.05) (Figure 7c). Compared to the vehicle control, ursolic acid significantly inhibited (*p* < 0.05) VEGF production by 15.18 ± 1.14%, which was statistically similar to the inhibitory potential of BS at 30 µg/mL (15.84 ± 4.54%) and DT-BS-01 at 5 µg/mL (12.21 ± 3.48%), indicating that BS and DT-BS-01 had a similar inhibitory potential to ursolic acid (Figure 7c). These findings indicate that BS and DT-BS-01 inhibited the production of VEGF at the active antiproliferative concentration of 30 µg/mL BS and 5 µg/mL DT-BS-01.

### 2.6. Ex Ovo Yolk Sac Membrane Assay (YSM)

**Figure 8:**
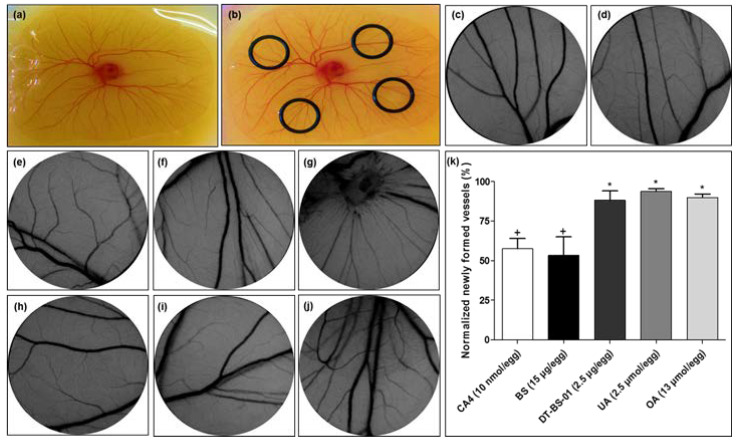
Images of (**a**) chick embryo, and (**b**) O-rings placed on the membrane for sample addition. Representative images of yolk sac membrane (YSM) treated for 24 h with (**c**) phosphate-buffered saline, (**d**) 0.3% DMSO (vehicle) control, (**e**) 3% DMSO (vehicle) control, (**f**) combretastatin A4 (10 nmol/egg), (**g**) *Buddleja saligna* extract (BS: 15 µg/egg), (**h**) isolated triterpenoid mixture (DT-BS-01: 2.5 µg/egg), (**i**) ursolic acid (2.5 µmol/egg) and (**j**) oleanolic acid (10 µmol/egg). (**k**) Normalized newly formed vessels (%) in YSM after treatment for 24 h. Data was expressed as mean ± SD (*n* = 2). * *p* < 0.05 indicates statistical difference when compared to the CA4 positive control (+), whereas (+) for BS, indicates statistically similar results to CA4. Statistical analysis was done using one-way ANOVA followed by Tukey’s Multiple Comparison Test.

The ex ovo YSM assay was used to determine the effect of BS and DT-BS-01 on angiogenesis using an in vivo model (Figure 8). BS at a concentration of 15 µg/egg was able to decrease the number of newly formed microvessels, with 53.34 ± 11.64% newly formed vessels, which was statistically similar to the positive control, combretastatin A-4 (CA4) at a concentration of 10 nM/egg, which showed 57.49 ± 6.48% newly formed vessels (Figure 8k). In a previous study, CA4 reduced blood vessels in a choriollantoic membrane (CAM) model by 26.8, 47.2 and 77.3% at 1, 5 and 10 nM/egg, respectively, through attenuation of the VEGF/VEGFR-2 pathway [34]. DT-BS-01 (at 2.5 µg/egg), UA (at 2.5 µM/egg) and OA (at 13 µM/egg), were statistically different (*p* < 0.05) compared to CA4 with 88.20 ± 5.79, 93.78 ± 1.71 and 89.89 ± 2.1% newly formed vessels, respectively (Figure 8k), suggesting that DT-BS-01, UA and OA did not inhibit angiogenesis at the tested concentrations.

### 2.7. Nanoparticle Synthesis

The synthesis of nanoparticles (Figure S6) was confirmed by ultraviolet-visible (UV-Vis) spectrometry, which determined the surface plasmon resonance (SPR) at 535 and 435 nm for BS-AuNPs and BS-AgNPs, respectively (Figures S7a and S8a). Additionally, BS was capable of reducing the Pd precursor to corresponding Pd nanoparticles (BS-PdNPs) as confirmed by the disappearance of the 415 nm absorption peak associated with Pd^II^ ions (Figure S9a). The colour change in the nanoparticles, where BS-AuNPs turned ruby-red, while BS-PdNPs and BS-AgNPs turned black and brown, respectively, further confirmed the synthesis of the nanoparticles (Figures S7a, S8a and S9a). The hydrodynamic size (nanoparticle core size including the phytochemical coating) was measured by dynamic light scattering (DLS), which was determined as 68.87 ± 1.0, 42.22 ± 1.0 and 94.70 ± 1.4 nm for BS-AuNPs, BS-AgNPs and BS-PdNPs, respectively (Figures S7e, S8e and S9c). Negative zeta potentials of −33.6 ± 1, −30.1 ± 1.4 and −16 ± 0.1 mV were determined for BS-AuNPs, BS-AgNPs and BS-PdNPs, respectively (Table 2). The stability of the BS-AuNPs, BS-AgNPs nanoparticles after 24 and 48 h in various buffer solutions was confirmed by the plasmon resonance peak at ~535 nm for BS-AuNPs and ~435 nm for BS-AgNPs (Figures S7b,c and S8b,c). DLS analysis confirmed the stability of BS-PdNPs in various buffer solutions (Figure S9d,e). The core size and morphology of BS-AuNPs, BS-AgNPs and BS-PdNPs nanoparticles were evaluated using transmission electron microscopy (TEM), which revealed core sizes of 16.8 ± 11.7, 14.5 ± 7.7 and 5.3 ± 2.9 nm, respectively. The synthesized nanoparticles were monodispersed and had a spherical shaped morphology (Figures S7d,e, S8d,e and S9b,c). The polydispersity index (PDI) of the synthesized nanoparticles was < 0.7 for each of the nanoparticles.

BS-AgNPs exhibited the most promising overall antiproliferative activity with the highest SI values of 2.34 and 2.35 when compared to HaCat and Raw 264.7 cell, respectively. This was followed by BS-PdNPs which revealed an SI value of 1.16 and 2.21, respectively. BS-AuNPs, however showed a significantly lower SI value (0.41) when compared to RAW 264.7 cells, suggesting its greater toxicity towards non-tumorigenic macrophages compared to the melanoma cells, however an SI of 1.70 was obtained when compared to HaCat cells. This was compared to the positive control, actinomycin D, which showed an SI value of 1.52 when compared to HaCat cells (Table 2).

## 3. Discussion

The antiproliferative activity of BS has been reported in two other studies. Bamuamba et al. (2008) [35] reported IC_50_ values > 100 µg/mL against Chinese Hamster Ovarian (CHO) cells for two fractions prepared from a hexane leaf partition, whereas Chukwujekwu et al. (2014) [36] reported an IC_50_ value of 1.7 ± 0.31 µg/mL for a hexane partition prepared from the leaves against CHO cells. UA and OA have been previously identified in the leaves of BS [37], and their anticancer properties have been extensively documented. In a study by Caunii et al. (2017) [38], UA inhibited the growth of SK-MEL-2 human melanoma cells (IC_50_: 58.43 µM), while the IC_50_ value of OA was >100 µM. Similarly, Mahmoudi et al. (2014) [39] showed that UA inhibited the growth of several melanoma cell lines with IC_50_ values of 33.09 ± 0.13, 26.25 ± 0.18, 18.15 ± 0.05, 19.45 ± 0.12 and 46.71 ± 1.28 µM on MM200, Mel-RM, Me4405, A375 and HFFF2 cells, respectively. A study by Oprean et al. (2018) [40] showed that UA induced a cytotoxic effect against A375 cells, while OA showed mild to moderate cytotoxicity. However, Ghosh et al. (2014) [41] found that OA had an IC_50_ value of 40.70 µM against A375 cells, while exhibiting negligible cytotoxicity against HaCat cells and PBMCs. Other compounds such as betulonic acid, betulone and spinasterol have been previously identified in the leaves of BS [42]. Findings from the hematoxylin and eosin staining suggest that both BS and DT-BS-01 induced apoptosis in UCT-MEL-1 and HaCat cells. Subsequent studies were conducted at the active antiproliferative concentrations, i.e., IC_50_ values of 30 µg/mL for BS and 5 µg/mL for DT-BS-01. 

OA has previously been reported to induce apoptosis in A375 cells by upregulating the expression of p53, Bax, cytochrome c, caspase 3 and cleaved PARP, while down regulating Bcl-2 [41]. These findings were confirmed in a study by Pratheeshkumar & Kuttan (2011) [43], where OA induced apoptosis in B16-F10 melanoma cells by upregulating p53, Bax, caspase-9 and caspase-3, and downregulating Bcl-2. Similarly, UA has been reported to induce apoptosis in A2058 melanoma cells [44]. Harmand et al. (2005) [45] reported that UA induced apoptosis in M4Beu melanoma cells through the mitochondrial intrinsic pathway by lowering transmembrane potential, increasing Bax expression, decreasing Bcl-2 expression and activating caspase-3. Additionally, Manu & Kuttan (2008) [46], demonstrated that UA induced apoptosis in B16F10 melanoma cells by upregulating p53 and caspase-3 while downregulating Bcl-2.

UA and OA have further been reported to inhibit COX-2 enzyme activity with IC_50_ values of 130 and 295 µM, respectively. Against the COX-1 enzyme, UA and OA showed IC_50_ values of 210 and 380 µM with an SI value of 0.6 and 0.8, respectively [47]. Additionally, UA inhibited phorbol 12−myristate 13−acetate (PMA)-mediated induction of COX-2 protein and synthesis of PGE2 as well as the expression of COX-2 mRNA, thus inhibiting COX-2 transcription in human mammary epithelial cells, while not affecting COX-1 [48]. UA significantly inhibited LPS—induced PGE2 production in murine macrophages (RAW 264.7), by 22.4, 25.1 and 65.6% at 1, 5 and 10 µM, respectively, and suppressed COX-2 expression by 9.3, 40 and 52.3%, respectively [49]. *Buddleja saligna* has not previously been reported to have an inhibitory potential against COX-2. Given the ability of both BS and DT-BS-01 to directly inhibit the COX-2 enzyme, one ought to evaluate whether these samples are capable of inhibiting COX-2 mRNA and protein expression in melanoma.

In addition there have been no previous reports on the inhibitory potential of BS against human inflammatory cytokines. OA, on the other hand, has been reported to significantly inhibit IL-6 levels in insulin-resistant human hepatoma (HepG2) cells [50] and inhibit the secretion of IL-6 from differentiating 3T3-L1 adipocytes [51]. UA suppressed IL-8 production in LPS-stimulated lung cancer (A549) cells with an IC_50_ value of 2.0 ± 0.14 µM, while OA inhibited IL-8 production at a concentration > 125 µM, which was also toxic to the cells, suggesting that the inhibition of IL-8 was related to the antiproliferative activity [52]. Additionally, UA significantly inhibited the production of IL-8 in IL-1β stimulated colon cancer (HL-29) cells [53]. A study by Manu & Kuttan (2008) [46], further showed that UA at a concentration of 50 µM, inhibited IL-6 production in B16F10 melanoma cells.

In a previous study, angiogenesis was induced in mice by injecting B16-F10 melanoma cells. Serum levels of VEGF increased from 70.78 ± 1.1 to 131.7 ± 18.86 pg/mL, after 24 h and ninth day collection, respectively, whereas tumor-bearing mice treated with UA (50 µmol/kg body weight) demonstrated a significant decrease in VEGF serum levels at 24 h collection (42.53 ± 0.53 pg/mL) and on the ninth day of collection (61.63 ± 1.2 pg/mL). VEGF levels in tumor-free mice were 16 ± 8.0 pg/mL [54]. In HUVEC cells treated with 20 ng/mL VEGF, OA was found to inhibit VEGF-induced phosphorylation of VEGF receptor 2 (VEGFR-2) in a dose-dependent manner. OA at 10 µM, inhibited approximately 50% of VEGFR-2 phosphorylation, suggesting that OA inhibited VEGF-induced VEGFR-2 activation. Additionally, OA inhibited VEGF-induced cell migration [55]. Furthermore, both OA and UA have been reported to decrease the number of vessels present in a CAM’s vascular network. Caunii et al. (2017) [38] found that OA (at 30 µM) was more effective than UA (at 30 µM) in reducing the vascular density and fine capillaries in several areas. In a melanoma (SK-MEL-2) CAM model, OA was able to reduce the angiogenic potential of the melanoma, while UA had no effect on the density of the capillaries within the CAM. In this same study, UA was shown to exhibit a more cytotoxic effect than OA against SK-MEL-2, suggesting that UA and OA in combination might trigger a synergistic or additive effect. A study by Cárdenas et al. (2004) [56], showed that a concentration of 50 µmol UA, inhibited angiogenesis in 100% of eggs, whereas a concentration of 20 µmol inhibited angiogenesis in 50% of eggs. Sohn et al. (1995) [57], reported that doses of 5 and 40 µg per CAM were required to obtain half—maximal inhibition (ID_50_).

Silver, palladium, and gold nanoparticles (NPs) were synthesized using BS and evaluated for antiproliferative effects against UCT-MEL-1, HaCat and RAW 264.7 cells. Padalia et al. (2015) [58], demonstrated the synthesis of AgNPs using a marigold flower extract and reported an SPR at 430 nm, that corroborates the BS-AgNPs results. Elia et al. (2014) [59], synthesized AuNPs using extracts from *Punica granatum* fruits and *Pelargonium graveolens* leaves and reported an SPR at 530 nm, comparable to that obtained for BS-AuNPs. The disappearance of the 415 nm absorption peak confirming that the Pd^II^ was reduced to its zero—valent (Pd^0^) state [60]. Due to the capping of the core by the excess phytochemicals in the reaction mixture, the core of the nanoparticles is anticipated to be significantly lower than the hydrodynamic size. The surface charge (zeta potential (ζ)) of the nanoparticles was determined to evaluate their stability in solution. The highly negative zeta potential demonstrates the repulsive forces that exist within the nanoparticles in solution, which prevents them from aggregating. Furthermore, BS-AgNPs exhibited the highest antiproliferative activity against UCT-MEL-1 cells, followed by BS-PdNPs and BS-AuNPs.

## 4. Materials and Methods

### 4.1. Materials

The VEGF ELISA kit, Dulbecco’s modified Eagle’s medium (DMEM), 0.25% trypsin-EDTA, phosphate—buffered saline (PBS), fetal bovine serum (FBS), antibiotics and PrestoBlue cell viability reagent were purchased from ThermoFisher Scientific (Johannesburg, South Africa). Sterile cell culture plates and flasks were obtained from Lasec SA (Pty) Ltd. (Midrand, South Africa). The BD^TM^ Cytometric Bead Array (CBA) Human Inflammatory Cytokine kit, BD Cytofix^TM^ fixation buffer and the BD Phosflow^TM^ permeation buffer were purchased from BD Biosciences (San Jose, CA, USA). The PGE_2_ ELISA kit was purchased from Biocom Biotech (Pty) Ltd. (Pretoria, South Africa). Sodium tetrachloroaurate (III) dehydrate (NaAuCl_4_) and silver nitrate (AgNO_3_) was procured from Alfa Aesar (Tewksbury, MA, USA). The Cell Proliferation Kit II (XTT) as well as all other chemicals and reagents, including actinomycin D (purity ≥ 95%), oleanolic acid (purity ≥ 97%), ursolic acid (purity ≥ 90%), human cyclooxygenase-2 enzyme, sodium tetrachloropalladate (II) (Na_2_PdCl_4_) and Folin—Ciocalteu phenol reagent were purchased from Sigma Chemicals Co. (St. Louis, MO, USA).

### 4.2. Cell Lines

Pigmented human melanoma (UCT-MEL-1), obtained from a metastatic lymph node of a patient at the Groote Schuur Hospital, Cape Town, South Africa, and human keratinocytes (HaCat), were kindly donated by the Department of Human Biology, University of Cape Town, South Africa. Raw 264.7 cells were purchased from Separations Scientific (Roodepoort, South Africa). Cell lines were maintained in DMEM containing 10% FBS and 1% penicillin (100 U/mL), streptomycin (100 µg/mL) and amphotericin B (250 µg/mL) under standard conditions in a humidified incubator (Thermo Scientific Forma, ThermoFisher Scientific, Johannesburg, South Africa) set at 5% CO_2_; 37 °C until 80% confluent, where after cells were sub-cultured using 0.25% trypsin-EDTA (0.1%).

### 4.3. Fertilized Eggs

Fertilized eggs were obtained from Pinot Valouro (Bombarral, Portugal). The ex ovo YSM was performed according to the Directive 2010/63/EU of the European Parliament and the council of 22 September 2010 on the protection of animals used for scientific purposes.

### 4.4. Preparation of Plant Extract

The ethanolic leaf extract was prepared as described previously, with slight modifications [61]. Leaves and stems of *Buddleja saligna* (Willd.) were collected in February 2015 from the Manie van der Schijff Botanical Gardens, University of Pretoria, South Africa. The plant material was identified by the curator, Mr Jason Sampson, and a voucher specimen (PRU 122167) was deposited in the HGWJ Schweickerdt Herbarium, Pretoria, South Africa. The plant material was shade dried (56.5% moisture loss) at room temperature and powdered using an IKA MF 10 universal grinder (Merck KGaA, Darmstadt, Germany). The powdered plant material (1.66 kg) was extracted using absolute ethanol (9 L) and left on a shaker (Labcon, Krugersdorp, South Africa) for 72 h. The extract was filtered through a Büchner funnel using Whatman no. 1 filter paper. The extraction and filtration procedures were repeated another two times with 5 L and 4 L of absolute ethanol, respectively. The solvent from the three extraction procedures were combined and evaporated under reduced pressure at 45 °C using a Büchi Rotavapor R-114 (Labotec, Midrand, South Africa) to obtain 200 mL of solvent. The remaining 200 mL of solvent was freeze dried for 2 weeks to obtain 240.68 g of dry extract (14.5% yield), which was kept at 4 °C until further use.

### 4.5. Liquid-Liquid Partitioning

The sequential partitioning into hexane and DCM was done according to a method previously described [42]. The crude ethanolic extract (160 g) was dissolved in 100% methanol (500 mL) followed by extraction with hexane (8 × 500 mL) in a separating funnel. The hexane layers were combined and evaporated under reduced pressure at 40 °C using a Büchi Rotavapor R-114 to obtain 11.45 g. The remaining methanol layer was concentrated in the same manner and the dried extract (145 g) was re-dissolved in distilled water (250 mL) by sonication for 30 min. The re-dissolved water partition was extracted with dichloromethane (DCM) (4 × 400 mL) in a separating funnel. The DCM layers were combined and dried under reduced pressure to obtain 22.12 g. The water partition was freeze dried for 1 week to obtain 40.29 g.

### 4.6. Bioassay-Guided Fractionation

The DCM partition (12 g) was re-dissolved in DCM (50 mL) and mixed with silica gel to form a slurry. The dried slurry was placed on a column packed with silica gel. The column was eluted with a mixture of hexane: DCM (100:0 to 0:100) followed by hexane: ethyl acetate (100:0 to 0:100) and ethyl acetate: methanol (100:0 to 0:100) of increasing polarity. A total of 66 major fractions were collected and pooled together according to similarity in thin-layer chromatographic (TLC) profiles. The major fractions were combined into 4 sub-fractions (M1, M2, M3 and M4). M4 showed the highest antiproliferative activity against UCT-MEL-1 cells (IC_50_: 13.08 ± 0.02 µg/mL) and therefore, was subjected to further isolation (Table 1). M4 (907 mg) was chromatographed on a Sephadex LH-20 column using DCM: methanol as the eluent from which 14 sub-fractions were collected and pooled together based on the TLC profiles. Sub-fraction 1–9 (410 mg) were combined and subject to silica gel chromatography with DCM: methanol at a 98:2 ratio as an eluent, which yielded an amorphous white powder, compound 1; DT-BS-01 (C1; 38 mg), which migrated as a single spot on the TLC plate. Upon identification of DT-BS-01 by ^1^H and ^13^C NMR (400 MHz Bruker Avance II; 5 mm BBO probe) spectroscopic data as well as COSY, HSQC, HMBC and LC-MS it was found to be a mixture of two pentacyclic triterpenoids; oleanolic acid and ursolic acid (Figures S1–S5). As these compounds are isomers, they were observed as one spot on the TLC plate.

### 4.7. LC-MS Analysis

According to tentative identification of DT-BS-01, the reference standards oleanolic acid and ursolic acid were used to confirm identification of DT-BS-01 using LC-MS analysis A Waters Acquity UPLC system with a binary solvent system (Waters Corporation, MA, USA) coupled to a Waters Synapt G2 mass spectrometry was used. Separation was performed on a kinetex^®^ 1.7 µm EVO C18, 2.1 mm × 100 mm column which was set at 40 °C and the flow rate was kept constant at 0.35 mL/min, with an injection volume of 7 µL. The mobile phase consisted of A: 0.1% formic acid in ultrapure water and B: methanol with 0.1% formic acid. A total run time of 25 min was used following a gradient elution method as follows: 20% B (0.0 min); 100% B (15–22 min); 20% B (23–25 min). The mass spectrometry (MS) was operated in positive and negative ESI resolution mode. Nitrogen gas was used as desolvation gas. MS data was acquired between 50 and 1200 *m*/*z*. The following parameters were set: Capillary voltages of 2600 V; sampling cone voltages of 30 V; extraction cone was 4 V; source temperature was 120 °C; desolvation temperature was 300 °C; desolvation gas 600 L/h; Cone Gas flow 10.0 L/h. Throughout all acquisitions, a 2 ng/µL solution of leucine enkephalin was used as the lockspray solution that was constantly infused at a rate of 5 µL/min through a separate orthogonal ESI probe so as to compensate for experimental drift in mass accuracy. The complete system was driven by Masslynx software.

### 4.8. Antiproliferative Activity

Antiproliferative activity was measured using the XTT Cell Proliferation Kit II and PrestoBlue^®^ cell viability reagent according to a previously described method [62]. Cells were seeded at a concentration of 1.0 × 10^5^ cells/well (100 µL) in 96−well plates and allowed to adhere for 24 h at 37 °C at 5% CO_2_. The extract, partitions and compounds were prepared at stock concentrations of 20 mg/mL (in DMSO), serially diluted, and added to the 96-well plates at final concentrations ranging from 3.125–200 µg/mL for the extracts and partitions and 0.781–100 µg/mL for the compounds. Synthesized nanoparticles were tested at concentrations ranging from 0.59–75 µg/mL (based on the calculated phenolic content). Controls included a 2% DMSO vehicle control, cells grown in media (untreated), a 0% control (no cells) and the positive control, actinomycin D at final concentration ranging from 3.9 × 10^−^^4^–0.05 µg/mL. Cells were incubated for a further 72 h with the respective samples and controls. Thereafter, 50 µL XTT (0.3 mg/mL) was added to the cells and incubated for 2 h where after the absorbance was measured at 490 nm (reference wavelength of 690 nm) using a BIO-TEK power-wave XS plate reader (A.D.P, Weltevreden Park, South Africa). Blank plates (for XTT viability) were included which were prepared in the same manner as mentioned above, without the additional of cells, to allow for color compensation of the samples. Each sample was tested in triplicate and the percentage cell viability was calculated using the below equation.

$$\%\;\mathrm{Cell}\;\mathrm{viability}=\frac{\mathrm{Abs}\;\mathrm{sample}}{\mathrm{Abs}\;\mathrm{control}}\times 100$$

where Abs control is the absorbance of (XTT + vehicle control) − (blank values of vehicle control) and Abs sample is the absorbance of (XTT + sample OR positive control) − (blank values of corresponding sample).

The antiproliferative activity of the nanoparticles was measured using PrestoBlue cell viability reagent, where 20 µL of PrestoBlue was added to all the wells after 72 h sample incubation, where after the cells were incubated for an additional 2 h. Fluorescence was measured at an excitation/emission of 560/590 nm using a Victor Nivo microplate reader (Perkin Elmer Inc., MA USA). The percentage cell viability was calculated using the below equation.

$$\%\;\mathrm{Cell}\;\mathrm{viability}=\frac{\mathrm{Flour}\cdot \;\mathrm{sample}-\mathrm{Flour}\;0\%\;\mathrm{control}}{\mathrm{Flour}\cdot \;\mathrm{DMSO}\;\mathrm{control}-\mathrm{Flour}\;0\%\;\mathrm{control}}\times 100$$

The fifty percent inhibitory concentrations (IC_50_) were calculated from the % cell viability using GraphPad Prism 4 software.

### 4.9. Cell Morphology-Light Microscopy (Hematoxylin and Eosin Staining)

Light microscopy (hematoxylin and eosin staining) was used to determine the quaitative effect of BS and DT-BS-01 on the morphology of UCT-MEL-1 and HaCat cells. Stock concentrations of BS and DT-BS-01 were prepared at 1 mg/mL (in 0.25% DMSO). BS and DT-BS-01 showed IC_50_ values of approximately 30 and 5 µg/mL, respectively, against UCT-MEL-1 cells, and approximately 60 and 20 µg/mL, respectively against HaCat cells, therefore these concentrations were selected for this experiment. Exponentially growing UCT-MEL-1 and HaCat cells were seeded at 1.0 × 10^5^ cells/well in a 6-well plate and incubated for 24 h at 37 °C at 5% CO_2_ to allow for cell adherence. Thereafter, cells were exposed to the BS (30 and 60 µg/mL) and DT-BS-01 (5 and 20 µg/mL), a 0.25% DMSO vehicle control, actinomycin D at 0.025 µg/mL and cells grown in media only (untreated) and incubated for a further 48 h. After 48 h, cells were fixed (in the 6−well plate) for 30 min in Bouin’s fixative. The fixative was discarded and replaced with 70% ethanol for 20 min, where after the cells were washed with distilled water. Hematoxylin was thereafter added for 20 min, followed by washing the cells with distilled water and 70% ethanol. The cells were then subject to eosin (1%) staining for 2 min followed by 2 times 5 min each washing procedures with 70, 96 and 100% ethanol. After staining, sterile PBS was added to all the wells and images were immediately taken using a light microscope (Zeiss Primovert, Zeiss, Johannesburg, South Africa) to observe morphological changes.

### 4.10. Cyclooxygenase-2 Inhibition

To determine the effect of BS and DT-BS-01 on the human recombinant cyclooxygenase-2 (COX-2) enzyme, a previously described method was used [63,64]. To each well of a 96−well plate, 180 µL of 100 mM TRIS buffer (pH 8.0) (co-factors: 5 µM porcine hematin, 18 mM L-epinephrine, 50 µM Na_2_EDTA) was added, followed by the addition of 5 µL COX-2 enzyme (0.5 units/well). Stock concentrations of the BS and DT-BS-01 were prepared at 10 mg/mL (in DMSO). Thereafter, 10 µL of BS and DT-BS-01 was added to the wells at final concentrations of 10–160 µg/mL (four-fold dilutions). Controls included a 5% DMSO vehicle control and a positive control Ibuprofen (0.4, 2 and 10 µM). After 5 min incubation, the reaction was initiated by adding 5 µL of 10 µM arachidonic acid and incubated at room temperature for a further 20 min. The reaction was inhibited by the addition of 10 µL of 10% formic acid. The PGE_2_ ELISA kit was used to quantify the production of PGE_2_ after the dilution of samples into a ratio 1:15 according to the manufacturer’s protocol (Cat. No. ADI-900-001) (Enzo Life Sciences, Inc., Farmingdale, New York, NY, USA). The absorbance, corresponding to the PGE_2_ concentration, was measured at 405 nm using a BIO-TEK power-wave XS plate reader. The percentage inhibition of PGE_2_ synthesis was calculated using the below equation.

$$\%\;\mathrm{Inhibition}\;\mathrm{of}\;{\mathrm{PGE}}_{2}=\frac{100-\left[{\mathrm{PGE}}_{2}\right]\;\mathrm{sample}}{\left[{\mathrm{PGE}}_{2}\right]\mathrm{control}}\times 100$$

where [PGE_2_]sample is the concentration of PGE_2_ (pg/mL) produced when treated with the sample OR positive control and [PGE_2_]control is the concentration of PGE_2_ (pg/mL) produced when treated with the 5% DMSO vehicle control. The IC_50_ values were calculated using GraphPad Prism 4 Software. 

### 4.11. Quantification of Human Inflammatory Cytokines

The quantification of cytokines expressed by UCT-MEL-1 cells was conducted in a similar manner as previously described [64]. The levels of cytokine production (Interleukin (IL)-8, -1β, -6, -10 & -12p70; and tumor necrosis factor alpha (TNF-α)) from cell supernatant were measured using the BD^TM^ Cytometric Bead Array (CBA) Human Inflammatory Cytokine kit according to the manufacturer’s protocol (Cat. No. 551811) (BD Biosciences, San Jose, CA, USA). Briefly, UCT-MEL-1 cells were seeded at a concentration of 1.0 × 10^5^ cells/well in a 24−well plate with complete medium at 37 °C and 5% CO_2_ to allow for cell adherence. After 24 h, the medium was discarded and replaced with fresh complete medium containing 1 µg/mL phytohemagglutinin (PHA) to stimulate cytokine production. Stock concentrations of BS and DT-BS-01 were prepared at 1 mg/mL (in 0.25% DMSO). Cells were treated with final concentrations of the BS (30 µg/mL) and DT-BS-01 (5 µg/mL). Controls included a 0.25% DMSO vehicle control and cells grown in media (untreated). After 20 h incubation, the cells were centrifuged at 980 rpm for 5 min to collect the cell free supernatant and analyze the concentration (in pg/mL) of cytokines using the BD^TM^ Accuri C6 cytometer and the FCAP Array™ Software V 3.0 (BD Biosciences, San Jose, CA, USA). The percentage inhibition was calculated using the following equation:

$$\%\;\mathrm{Inhibition}=100-\frac{\left[\mathrm{cytokine}\right]\mathrm{sample}}{\left[\mathrm{cytokine}\right]\mathrm{medium}}\times 100$$

where [cytokine]medium is the concentration (pg/mL) of the cytokine expressed in cells which contained medium only (untreated) and [cytokine]sample is the concentration (pg/mL) of the cytokine expressed in cells which contained the sample or DMSO.

### 4.12. Quantification of In Vitro VEGF

Quantification of VEGF production was done in a similar manner using an in vitro cell-based assay as previously described [65]. Exponentially growing UCT-MEL-1 and HaCat cells were seeded at a concentration of 1.0 × 10^5^ cells/well in a 24−well plate with complete medium at 37 °C and 5% CO_2_ for 24 h to allow for cell adherence. After 24 h, the medium was replaced with fresh complete medium. Stock concentrations of BS and DT-BS-01 were prepared at 1 mg/mL (in DMSO). The cells were treated with final concentrations of BS (30 µg/mL) and DT-BS-01 (5 µg/mL). Controls included a 0.15% DMSO vehicle control, cells grown in medium (untreated) and cells exposed to the positive control, 6 µg/mL (~13 µM) ursolic acid. After 6 h of treatment, the plates were centrifuged at 980 rpm for 10 min and the cell free supernatant collected for quantification of VEGF using the VEGF human ELISA kit (Novex^®^ Cat # KHG0111) (ThermoFisher Scientific, Johannesburg, South Africa) according to the manufacturer’s protocol, where absorbance was measured at 450 nm. Concentrations of VEGF were calculated from the standard curve using a linear equation. Percentage inhibition was calculated using the following equation:

$$\%\;\mathrm{Inhibition}=100-\frac{\left[\mathrm{VEGF}\right]\mathrm{sample}}{\left[\mathrm{VEGF}\right]\mathrm{medium}}\times 100$$

where [VEGF]medium is the concentration (pg/mL) of VEGF expressed in cells which contained medium only (untreated) and [cytokine]sample is the concentration (pg/mL) of VEGF expressed in cells which contained the sample or DMSO. Cell viability was further determined using XTT (0.3 mg/mL) to ascertain that inhibition was not due to cell death.

### 4.13. Ex Ovo YSM

The YSM was used to determine the effect of BS and DT-BS-01 on angiogenesis [66,67,68,69,70,71,72], with modifications [23]. Three days after fertilization, eggs were incubated for 72 h at 37 °C and 90% (*v*/*v*) relative humidity. During the incubation, eggs were gently turned twice a day to prevent adherence of the yolk sack to the shell. Thereafter, the eggs were opened into weighing boats (L89 × W89 × H25 mm) with the yolk sacs and blood vessels facing upwards. Weighing boats, with the same dimensions and with holes punctured in the sides, were used to cover the opened eggs. Once the opened eggs had stabilized for an additional 24 h at 37 °C and 90% (*v*/*v*) relative humidity, four sterilized silicone O-rings, with an internal diameter of 8 mm, were placed on the blood vessels. To each O-ring, 40 µL of sample was added, of which each egg contained at least one control. The controls included PBS and two vehicle treated controls (0.3 and 3% DMSO), where 0.3% was the vehicle for the compounds and 3% was used for BS. Samples included BS (15 µg/egg), DT-BS-01 (2.5 µg/egg), oleanolic acid (13 µmol/egg), ursolic acid (2.5 µmol/egg) and the positive control, combretastatin A4 (10 nmol/egg). After the addition of the samples to the O-rings, the eggs were incubated for a further 24 h at 37 °C and 90% (*v*/*v*) relative humidity. Images of each of the O-rings were taken at 0 and 24 h of incubation with the samples and controls using a digital USB microscope camera (Opti-Tekscope OT−V1). Images were analyzed using the Fiji ImageJ Software with the Analyze Skeleton plugin. The number of newly formed vessels was calculated by normalizing the number of newly formed vessels in the sample treated YSM to those of the 0.3 and 3% DMSO (vehicle) control.

### 4.14. Synthesis of Nanoparticles (Gold (Au), Palladium (Pd) and Silver (Ag)

*Buddleja saligna* dried powdered material (400 mg leaves and stems) was weighed into a 100 mL flaks followed by the addition of 40 mL distilled water (dH_2_O). The solution was heated to 90 °C for 5 min to allow for the extraction of polyphenols and proanthocyanidins. After the solution had cooled, it was centrifuged at 2000 rpm for 5 min. Thereafter, 6 mL of the collected supernatant was added to a 30 mL vial followed by the addition of the 0.1 M metal precursor solution (NaAuCl_4_, Na_2_PdCl_4_ and AgNO_3_, respectively). The solution was continuously stirred overnight at room temperature to optimize total reduction of the metal salts to their respective nanoparticle (BS-AuNPs, BS-AgNPs and BS-PdNPs) (Figure S6). The nanoparticles were centrifuged at 2000 rpm for 5 min, where after the supernatant was discarded to remove unbound compounds. The nanoparticles were reconstituted in dH_2_O for further use.

### 4.15. Characterization of Nanoparticles

Ultraviolet-visible (UV-vis) spectroscopy was used to determine the characteristic surface plasmon resonance (SPR) of the respective nanoparticles. The samples (200 µL) were added to 800 µL in a quartz cuvette. The absorbance of each sample was read at a wavelength ranging between 200–800 nm using a Cary 60 UV-Vis spectrophotometer (Agilent Technologies, Santa Clara, CA, USA). The same sample preparations were used to measure the hydrodynamic size and the zeta potential of the respective nanoparticles using a Zetasizer NanoSeries (Nano-ZS90) (Malvern Panalytical, Westborough, MA, USA). The average size of the respective nanoparticles was determined by obtaining transmission electron microscope (TEM) images on a JOEL 1400 TEM operating at 120 kV. The TEM samples were prepared by adding 5 µL of the respective nanoparticles on a carbon-coated copper grid and allowed to dry.

### 4.16. In Vitro Stability of Nanoparticles

The in vitro stability of the respective nanoparticles was evaluated in the presence of various biological buffer solutions to mimic in vivo conditions. The samples (1 mL) were added to glass vials containing 0.5 mL aqueous solutions of 5% NaCl, 0.5% cysteine, 0.2 M histidine, 0.5% human serum albumin (HSA), 0.5% bovine serum albumin (BSA) and PBS solutions at pH 5, 7 and 12, respectively. The stability was measured by monitoring the UV absorbance over a 24 and 48 h incubation period using a Cary 60 UV−Vis spectrophotometer. The stability of BS-PdNPs was monitored by DLS analysis since PdNPs do not exhibit an absorption peak.

### 4.17. Total Phenolic Content of Nanoparticle Solutions

The total phenolic content of the respective nanoparticles was determined using the Folin—Ciocalteu method. The samples (500 µL) were added to a 2 mL microcentrifuge tube followed by the addition of 500 μL 1/10 dilution Folin–Ciocalteu phenol reagent and 1 mL of 7.5% (*w*/*v*) sodium carbonate solution (Na_2_CO_3_). The tube was incubated in a Microtube Thermal Mixer (ThermoFisher Scientific, Waltham, MA, USA) at 30 °C for 30 min, where after the absorbance was measured at 760 nm using a microplate reader. The concentration of total phenolic content was expressed as gallic acid equivalence (GAE) in µg/mL.

### 4.18. Statistical Analysis

Results are reported as mean ± SD (or SEM). Samples were tested in duplicate or triplicate with at least 2 independent experiments (see Section 2), unless otherwise stated. Statistical analysis was done using one-way analysis of variance (ANOVA) followed by student’s *t*-test (unpaired), or Dunnett’s or Tukey’s Multiple Comparison Test using the GraphPad Prism statistical software. * *p* < 0.05; ** *p* < 0.01 and *** *p* < 0.001 indicated statistical significance compared to the control (+).

## 5. Conclusions

Herein, we determined that *Buddleja saligna* (BS) and the triterpenoid mixture (DT-BS-01) should be considered for preclinical studies (toxicity studies and an animal melanoma model) due to the significant antiproliferative activity demonstrated against melanoma cells. Subsequently, both these samples have the potential to inhibit several pro-angiogenic factors, the most important of which is VEGF, which is required to stimulate angiogenesis for metastasis to occur. Additionally, utilizing the ex ovo YSM assay, BS was able to significantly inhibit the formation of new blood vessels demonstrating its anti-angiogenic efficacy in an in vivo model. Furthermore, due to the antiproliferative activity and noteworthy SI values, BS-AgNPs and BS-PdNPs should be explored for future anti-angiogenic investigations. Future studies should further include determining the antiproliferative potential of BS, DT-BS-01, BS-AgNPs and BS-PdNPs, against several other melanoma cell lines as well as primary non-tumorigenic melanocytes and to quantitatively determine apoptosis using the TUNEL assay. This is the first report on the antiproliferative and anti-angiogenic activities of BS against cancer cell lines. Moreover, the compound mixture (DT-BS-01), which consists of ursolic acid and oleanolic acid, has never been reported in combination for its antiproliferative and anti-angiogenic activities.

## 6. Patents

D.T. and N.L. have filed a patent application related to the technology described in this article.

## Figures and Tables

**Table 1:** Antiproliferative activity of *Buddleja saligna* ethanolic extract, partitions, major fractions and compounds against melanoma (UCT-MEL-1) and non-tumorigenic keratinocytes (HaCat).

Sample	UCT-MEL-1 Cells	HaCat Cells	Selectivity Index
	IC_50_ ± SD (µg/mL)		
*Buddleja saligna* ethanolic extract	33.80 ± 1.02	54.38 ± 8.55	1.64
Hexane partition	109.92 ± 20.05	− ^1^	NA ^2^
Dichloromethane (DCM) partition	15.72 ± 1.34	−	NA
Water partition	57.69 ± 5.28	−	NA
M1 fraction of DCM partition	>200	−	NA
M2 fraction of DCM partition	28.89 ± 3.61	−	NA
M3 fraction of DCM partition	24.10 ± 3.07	−	NA
M4 fraction of DCM partition	10.73 ± 3.40	−	NA
DT-BS-01 subfraction of M4	5.45 ± 0.19	27.59 ± 2.86	5.06
Ursolic acid	2.31 ± 0.54	2.32 ± 0.52	1.01
Oleanolic acid	13.08 ± 3.03	16.84 ± 1.32	1.29
Actinomycin D	2.59 × 10 ^3^ ± 4.85 × 10^−4^	5.57 × 10^−3^ ± 2.50 × 10^−4^	1.52

**^1^** Not tested, ^2^ NA—not applicable.

**Table 2:** Physiochemical data parameters and antiproliferative activity of synthesized nanoparticles.

Sample	Hydrodynamic Size (nm)	PDI ^1^	Zeta Potential (mV)	TEM Size (nm)	Surface Coating (nm)	Total Phenolic Content (µg/mL GAE ^2^)	UCT-MEL-1 Cells	HaCat Cells	RAW 264.7 Cells
							IC_50_ ± SD (µg/mL)		
BS ^3^-AuNPs ^4^	68.87 ± 1.0	0.3	−33.6 ± 1.0	16.8 ± 11.7	52.1	182.7	42.72 ± 2.07	72.81 ± 4.89	17.49 ± 2.15
BS-AgNPs	42.22 ± 1.0	0.5	−30.1 ± 1.4	14.5 ± 7.7	27.7	292.4	16.00 ± 1.92	37.49 ± 0.19	37.54 ± 0.15
BS-PdNPs	94.70 ± 1.4	0.3	−16 ± 0.1	5.3 ± 2.9	89.4	165	33.74 ± 7.4	38.97 ± 0.42	74.57 ± 1.90

^1^ PDI—polydispersity index, ^2^ GAE—Gallic acid equivalent, ^3^ BS—*Buddleja saligna* extract, ^4^ NPs—nanoparticles.

## Data Availability

Data is contained within the article and Supplementary Materials.

## Supplementary Materials

The following supporting information can be downloaded at: https://www.mdpi.com/article/10.3390/ph15121497/s1, Figure S1: Representative (a) ^1^H-NMR (Methanol-d_4_, 400 MHz) and (b) ^13^C-NMR (Methanol-d_4_, 100 MHz) spectra of DT-BS-01; Figure S2: Extracted-ion chromatogram (XIC) of *m*/*z* 455.35 in negative ionization mode of LC-MS analysis of (a) DT-BS-01 (b) Oleanolic acid and (c) Ursolic acid; Figure S3: Extracted-ion chromatogram (XIC) of *m*/*z* 479.35 in positive ionization mode of LC-MS analysis of (a) DT-BS-01 (b) Oleanolic acid and (c) Ursolic acid; Figure S4: Negative ionization mode mass spectra of (a) DT-BS-01 (b) Oleanolic acid and (c) Ursolic acid; Figure S5: Positive ionization mode mass spectra of (a) DT-BS-01 (b) Oleanolic acid and (c) Ursolic acid; Figure S6: Synthesis process of nanoparticles using *Buddleja saligna*; Figure S7: Characterization of BS-AuNPs. (a) UV-Vis absorption spectra. (b) *In vitro* stability in buffer solutions after 24 h. (c) *In vitro* stability in buffer solutions after 48 h. (d) Transmission electron micrograph. (e) Particle size distribution; Figure S8: Characterization of BS-AgNPs. (a) UV-Vis absorption spectra. (b) *In vitro* stability in buffer solutions after 24 h. (c) *In vitro* stability in buffer solutions after 48 h. (d) Transmission electron micrograph. (e) Particle size distribution; Figure S9: Characterization of BS-PdNPs. (a) UV-Vis absorption spectra. (b) Transmission electron micrograph. (c) Particle size distribution (d) *In vitro* stability in buffer solutions after 24 h. (e) *In vitro* stability in buffer solutions after 48 h.
