# Supplementary material for: Antiproliferative Activity of Buddleja saligna (Willd.) against Melanoma and In Vivo Modulation of Angiogenesis
Source: Pharmaceuticals (Basel). 2022 Nov 30;15(12):1497. doi: 10.3390/ph15121497 (PMC9782150; doi:10.3390/ph15121497)
Supplement: Supplementary file 1 [file pharmaceuticals-15-01497-s001.zip › pharmaceuticals-1995830-supplementary.pdf]

Supplementary Information for
Antiproliferative activity of *Buddleja saligna* (Willd.) against melanoma and *in vivo* modulation of angiogenesis

Danielle Twilley[1], Velaphi C. Thipe[2], Navneet Kishore[1], Pierce Bloebaum[3], Catarina Roma-Rodrigues[4], Pedro V. Baptista[4], Alexandra R. Fernandes[4], Mamoalosi A. Selepe[5], Lenka Langhansova[6], Kattesh Katti[1,2,7,8*], Namrita Lall[1,9,10,11*]

This PDF file includes:

Figures S1 to S9

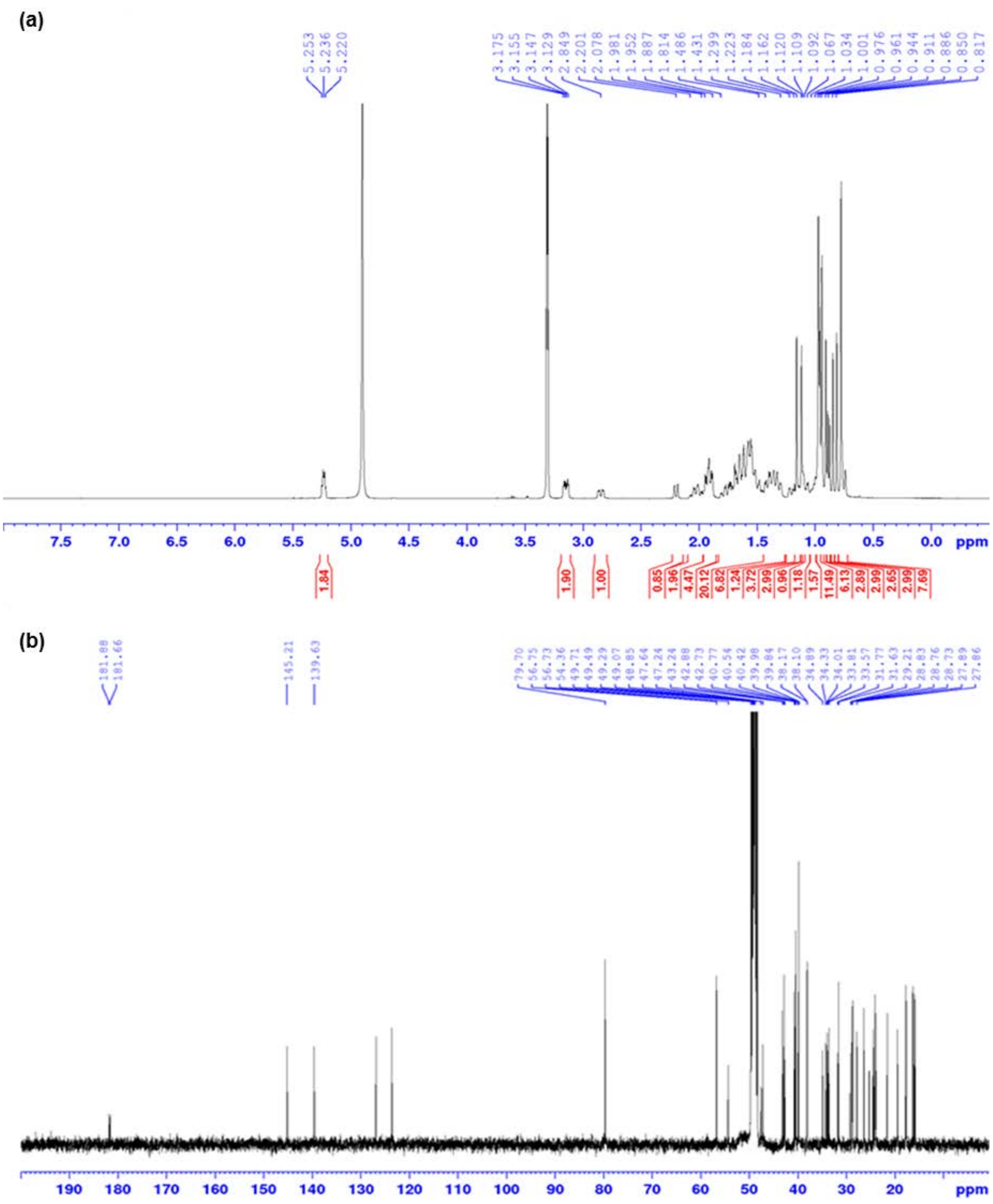

Figure S1. Representative (**a**) ^{1}H-NMR (Methanol-d$_4$, 400 MHz) and (**b**) ^{13}C-NMR (Methanol-d$_4$, 100 MHz) spectra of DT-BS-01

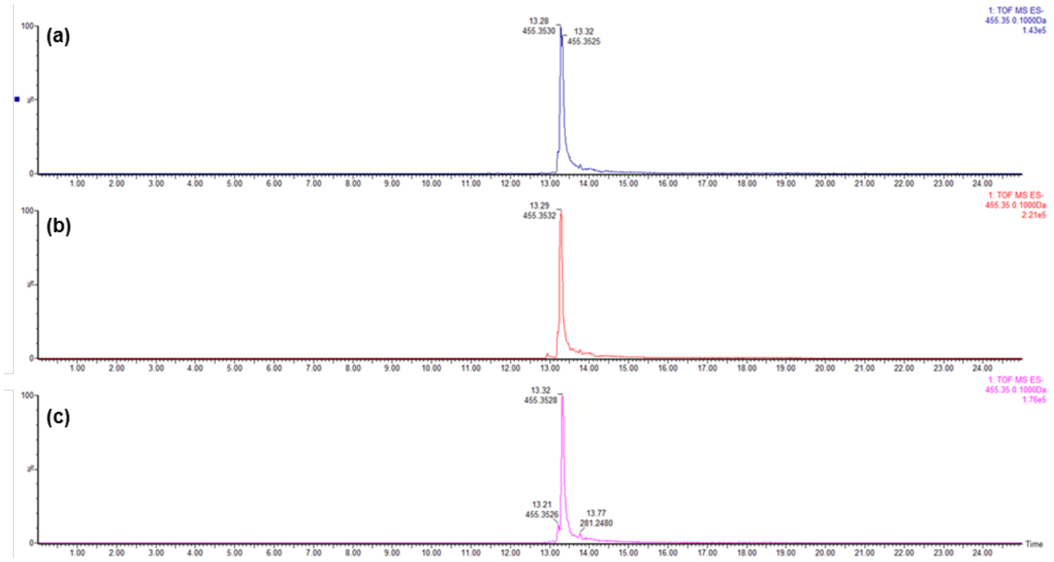

Figure S2. Extracted-ion chromatogram (XIC) of m/z 455.35 in negative ionization mode of LC-MS analysis of (**a**) DT-BS-01; (**b**) Oleanolic acid and (**c**) Ursolic acid

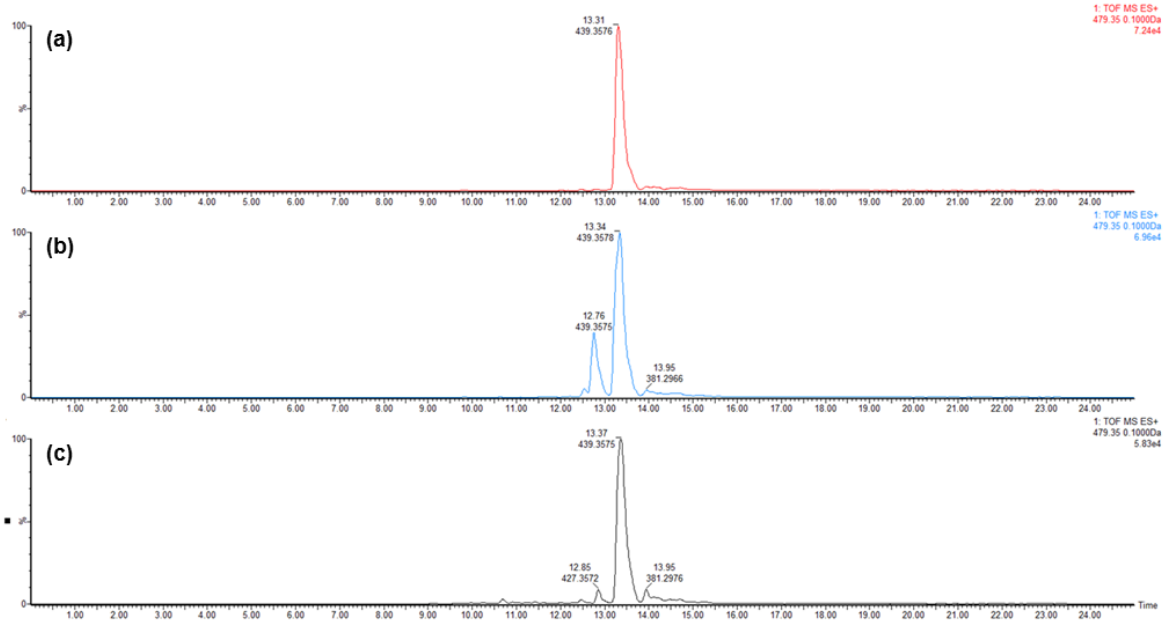

Figure S3. Extracted-ion chromatogram (XIC) of m/z 479.35 in positive ionization mode of LC-MS analysis of (**a**) DT-BS-01 (**b**) Oleanolic acid and (**c**) Ursolic acid

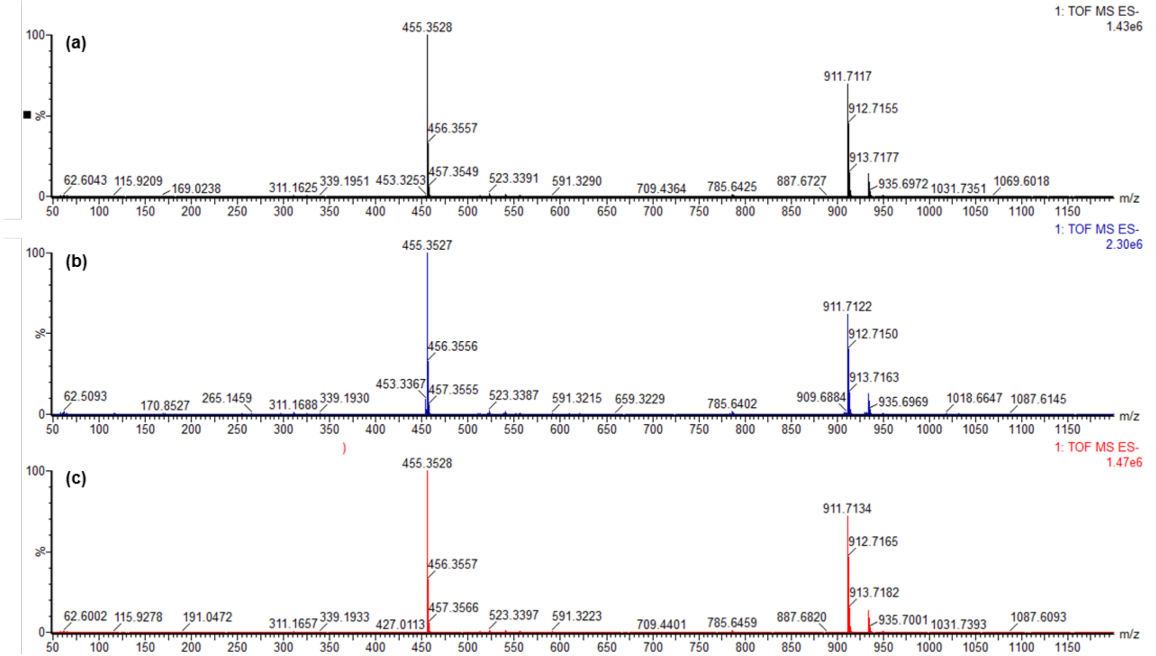

Figure S4. Negative ionization mode mass spectra of (**a**) DT-BS-01; (**b**) Oleanolic acid and (**c**) Ursolic acid

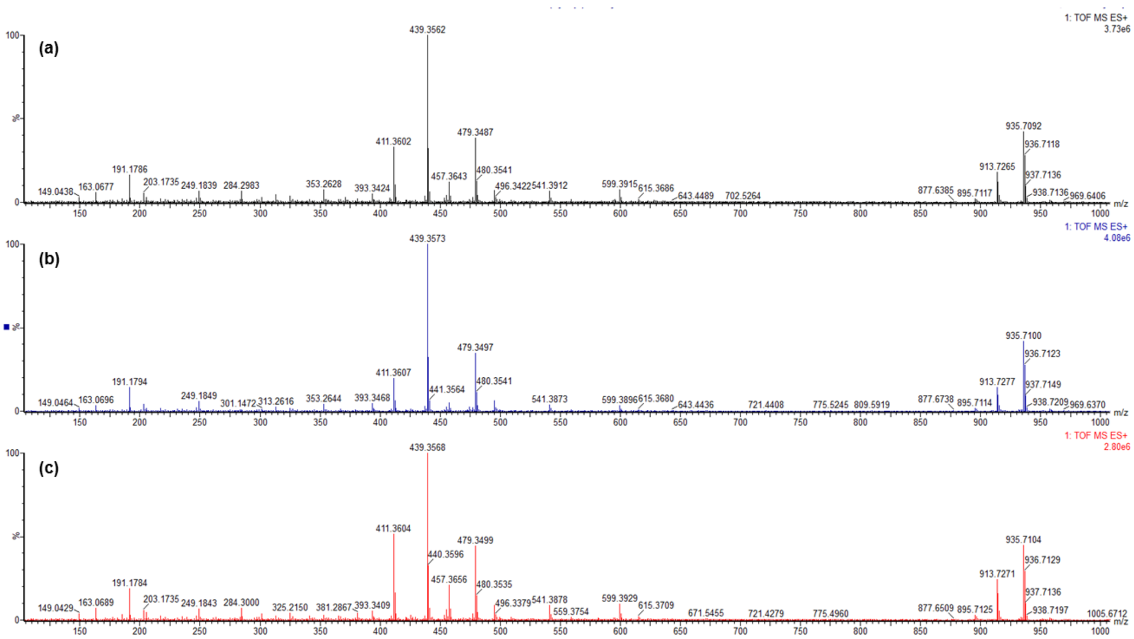

Figure S5. Positive ionization mode mass spectra of (**a**) DT-BS-01; (**b**) Oleanolic acid and (c) Ursolic acid

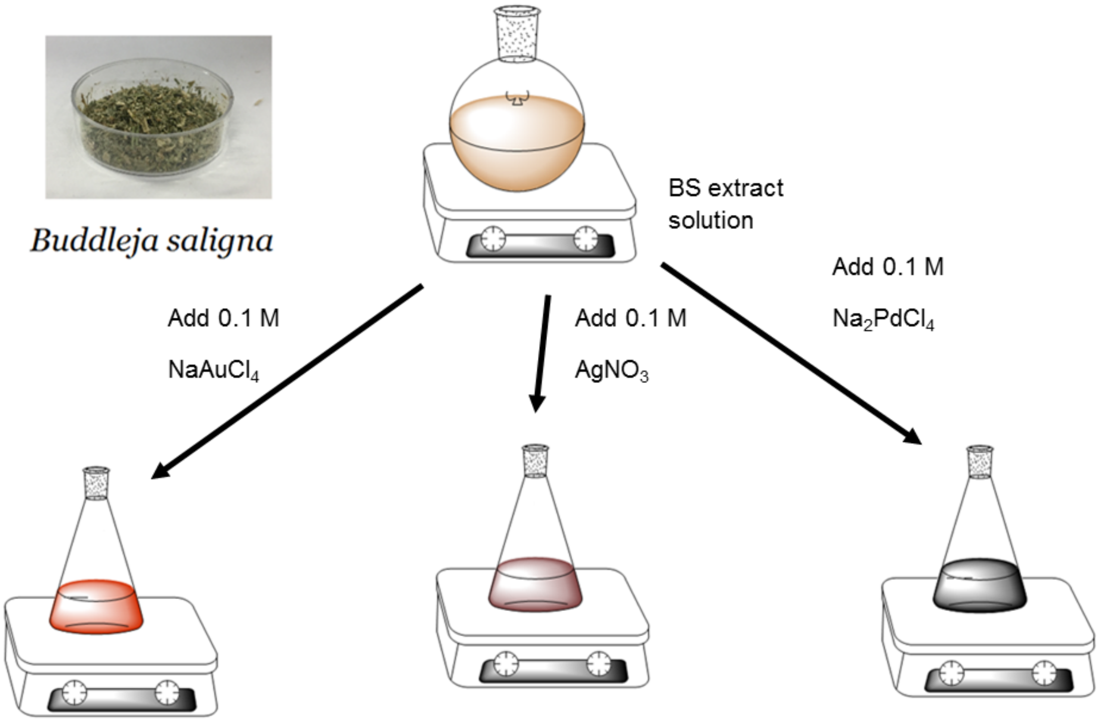

Figure S6. Synthesis process of nanoparticles using *Buddleja saligna*

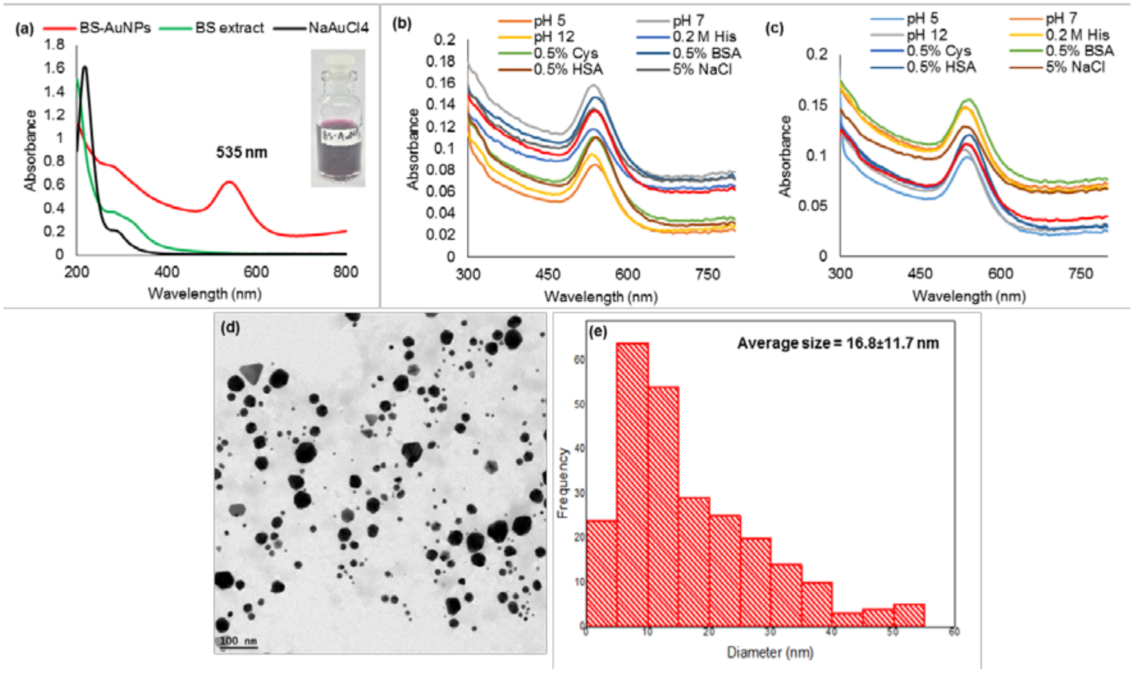

Figure S7. Characterization of BS-AuNPs. (**a**) UV-Vis absorption spectra; (**b**) *In vitro* stability in buffer solutions after 24 h; (**c**) *In vitro* stability in buffer solutions after 48 h; (**d**) Transmission electron micrograph; (**e**) Particle size distribution

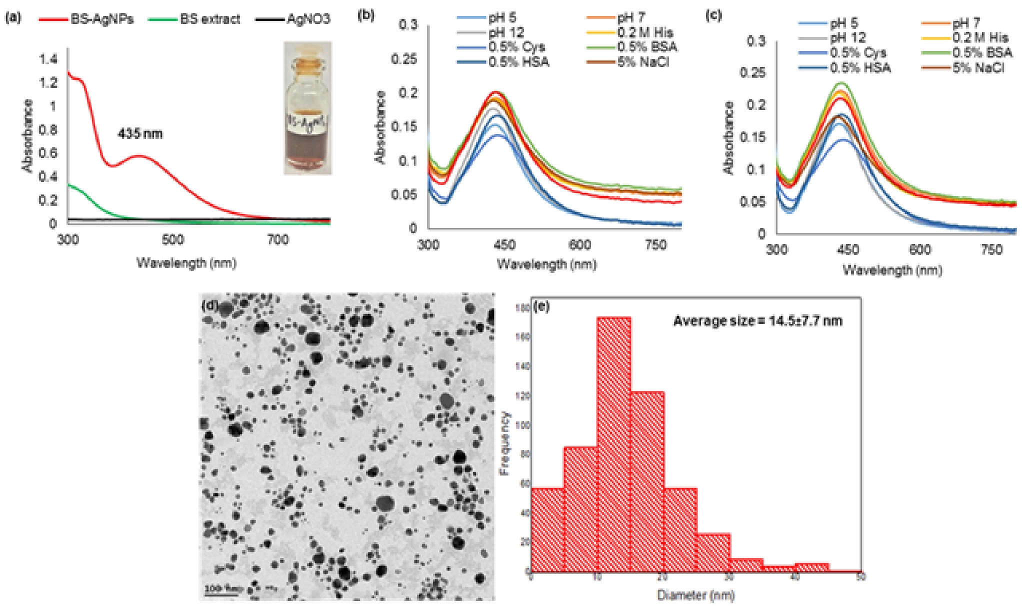

Figure S8. Characterization of BS-AgNPs. (a) UV-Vis absorption spectra. (b) *In vitro* stability in buffer solutions after 24 h. (c) *In vitro* stability in buffer solutions after 48 h. (d) Transmission electron micrograph. (e) Particle size distribution

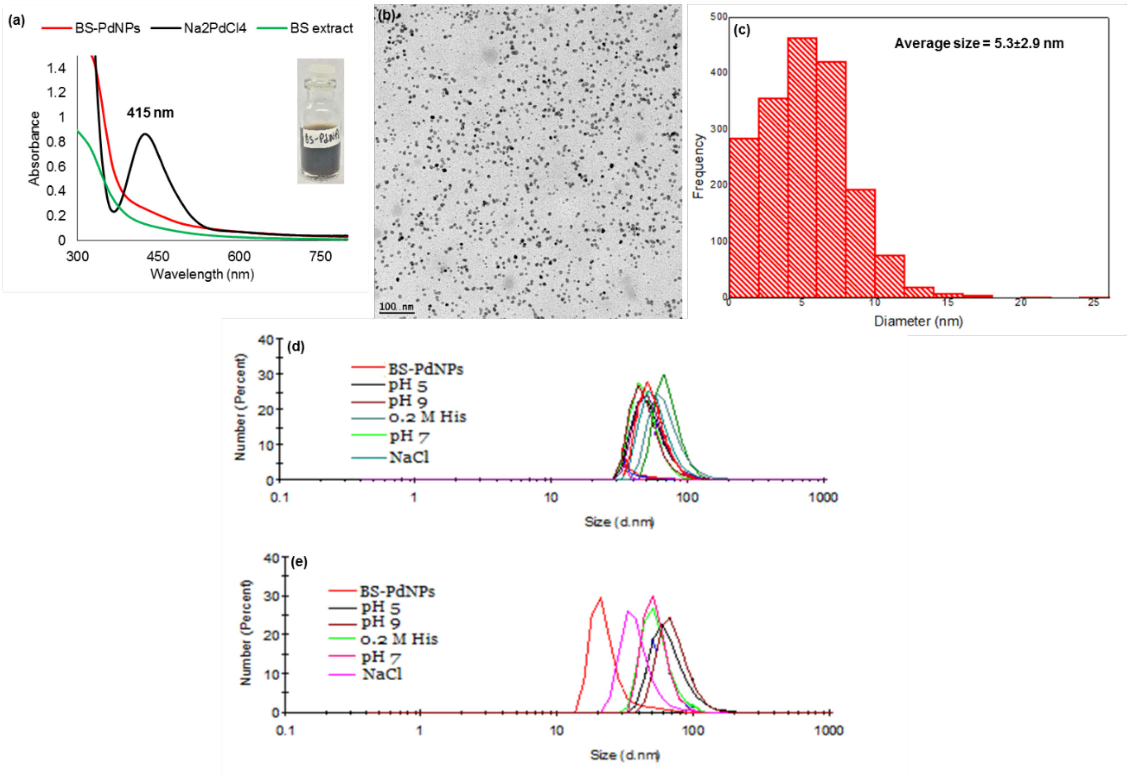

Figure S9. Characterization of BS-PdNPs. (**a**) UV-Vis absorption spectra; (**b**) Transmission electron micrograph; (**c**) Particle size distribution; (**d**) *In vitro* stability in buffer solutions after 24 h; (**e**) *In vitro* stability in buffer solutions after 48 h
